# Extended use of the modified Berlin Definition based on age-related subgroup analysis in pediatric ARDS

**DOI:** 10.1007/s10354-018-0659-6

**Published:** 2018-09-19

**Authors:** Michael Hermon, Sophia Dotzler, Jennifer Bettina Brandt, Wolfgang Strohmaier, Johann Golej

**Affiliations:** 1 Division of Neonatology, Pediatric Intensive Care and Neuropediatrics, Medical University of Vienna, Währinger Gürtel 18–20, 1090 Vienna, Austria; 2 Wilheminenspital, Vienna, Austria; 3 Institut für molekulare Biowissenschaften, Karl Franzens University Graz, Graz, Austria

**Keywords:** Pediatric ARDS, Children, Berlin Definition, ARDS supportive treatment, SpO2/FiO2 ratio

## Abstract

**Background:**

Pediatric acute respiratory distress syndrome (pARDS) is a rare but very severe condition. Management of the condition remains a major challenge for pediatric intensive care specialists.

**Objective:**

To perform a descriptive assessment of pARDS based on the modified Berlin Definition by using the SpO_2_/FiO_2_ ratio in order to establish an extended patient registry divided into age-related subgroups.

**Methods:**

The data of all children on mechanical ventilation for respiratory failure admitted between 2005 and 2012 were reviewed retrospectively for this study. The age of patients ranged from newborns >37 weeks, up to children <18 years. Inclusion criteria were based on the modified Berlin Definition of pARDS. The following data were collected: demographic data, primary diagnosis, ventilation settings, and use of supportive treatment, in addition to mechanical ventilation (inhaled nitric oxide, surfactant, corticosteroids, prone positioning, and extracorporeal membrane oxygenation).

**Results:**

In all, 93 children where included: 35% were newborns, 29% infants, 24% toddlers, and 12% school children; 66% were male and 34% were female patients. The most common primary diagnosis was viral pneumonia (21%) and 55% of the children were diagnosed with severe ARDS. The median duration of stay on the pediatric intensive care unit was 16 days (10/27). In total, 66 children (71%) had direct lung injury and 18 (19%) had indirect lung injury. More than 80% of all children needed more than one supportive care therapy. The overall survival rate was 77%.

**Conclusion:**

This study is a valuable report about pediatric patients with ARDS and allows for an important extension of the application of the modified Berlin Definition in all age groups.

## Introduction

Acute respiratory distress syndrome (ARDS), as first described by Ashbaugh et al. in 1967 [1], remains a therapeutical challenge for intensive care specialists. Pediatric ARDS (pARDS) can be described as a rare disease with an incidence of 2–12/100,000 per year. The low number of patients with pARDS makes it a challenging task to conduct clinical trails with conclusive results [2, 3]. At present, the overall mortality rate in pARDS is approximately 24% [4]. Over many years, pediatric intensive care specialists have used the American–European Consensus Conference (AECC) definition of ARDS for clinical care, research, and prognosis [5]. Limitations of the AECC definition of ARDS have recently been addressed by the Berlin Definition (BD) for adult patients, but pediatric-specific considerations were not included in these definitions [5, 6]. Although there are similarities in the pathophysiology of ARDS in adults and children, pediatric-specific therapies, comorbidities, and differences in outcome demand a more specific definition for pARDS patients [7]. In this context, a major limitation of the adult BD is the necessity for invasive measurement of arterial blood gas. Pulse oximetry increasingly prevents the use of arterial blood gas measurement in children, and therefore a definition requiring direct blood gas analysis may underestimate the prevalence of pARDS. Moreover, recent evidence shows that arterial catheters are an under-recognized source of infection and this may continue to contribute to a shift in practice patterns away from the routine use of such arterial catheters [8] and consequently PaO_2_ measurements. Several studies have demonstrated that the SpO_2_/FiO_2_ ratio (S/F ratio) can be used instead of the PaO_2_/FiO_2_ (P/F ratio) when the SpO_2_ is <97% [9–12].

In October 2013, De Luca et al. published a multicenter study with the main goal of investigating the validity of BD in infancy and early childhood [13]. Our study attempts to extend the systematical data collection and provide a more detailed description of pARDS patients divided into age-related subgroups (full-term newborns 0–28 days, infants 1–12 months, children 1–6 years, children 6–18 years). ARDS etiology (direct and indirect lung injury), severity of ARDS according to the modified BD, and five types of supportive treatment options were also investigated: inhaled nitric oxide (iNO), surfactant, corticosteroids, prone position, and extracorporeal membrane oxygenation (ECMO).

The objective of this study was to describe a cohort of patients with pARDS using the modified BD with the S/F ratio in order to establish an extended patient registry divided into age-related subgroups. A further aim of this study was to provide feedback to clinicians about their daily work concerning the quality of treatment and possible options for the improvement of treatment in the future.

## Patients and methods

After the approval of the ethics committee (Medical University of Vienna; Ethic Nr. 1860/2012) the patient records of all children admitted to our pediatric intensive care unit (PICU) between 2005 and 2012 were screened for eligibility. Because of the retrospective character of this study, informed consent was waived and the study was performed in accordance with the Declaration of Helsinki.

The data of 219 children—aged between full-term newborns and <18 years—with respiratory failure according to the modified BD of ARDS [6] were reviewed for inclusion in the study. Only patients with complete records were included. The 93 patients finally included were divided into three groups according to the ARDS etiology, direct and indirect lung injury, and a third group of ARDS patients with unknown etiology. Preterm newborns with a corrected age of >38 weeks of gestation at the time of admission were not excluded from the study. The following data were extracted from patient files: age (years), gender, weight (kg), duration of PICU stay (days). Primary diagnosis, relevant secondary diagnosis, and chronic or congenital diseases or developmental disorders were noted. The modes and duration of mechanical ventilation (MV) and ventilator settings were recorded. Oxygenation was evaluated by recording arterial blood gas samples and SpO_2_. If arterial blood gas samples were not available, the S/F ratio was used as a surrogate for P/F ratio [10–12]. Additional supportive treatment beside MV, such as iNO, surfactant, corticosteroids, prone positioning, and ECMO were noted. Outcomes were defined as survival of PICU stay.

Chest radiographs and levels of oxygenation were collected on day 1, day 3, and day 7 of PICU stay. However, these radiographs were not available for all patients for each of the 3 days, and thus patients were included if at least one chest radiograph was available. The chest radiographs were anonymized and examined by an independent radiologist.

Data analysis was performed using IBM SPSS Statistics Version 21.0 (IBM Deutschland GmbH, Ehningen, Germany). For statistical analysis, the chi-square test or Fisher’s exact test and independent-samples Kruskal–Wallis test were used. Results were accepted as statistically significant at *p* < 0.05. Data are presented as median and interquartile ranges or as percentages.

## Results

During the study period 2005–2012, 219 patient records were screened for eligibility criteria and 93 children were included in the study. We excluded 126 patients owing to missing data or chest radiographs. Table 1 shows the demographic data of all study patients. Because of the heterogeneity of the patient collective with a range in age from 0 days to 16.8 years, patients were divided into age-related subgroups to account for age-specific conditions and differences. The subgroup of school children was not divided any further owing to the small number of patients of this age. Seven patients (7%) had a history of preterm birth but had reached a corrected age of >38 weeks of gestation at admission and therefore were included.

**Table 1:** Demographic data of study patients

Total number of patients; *N* (%)	93 (100)	**–**
Age in years; median (IQR)	0.3 (0–16.8)	**–**
Gender: M/F; *N* (%)	61 (66)/32 (34)	**–**
History of preterm birth; *N* (%)	7 (7)	**–**
PICU stay in days; median (IQR)	16 (10–27)	**–**
Duration of mechanical ventilation; median (IQR)	12 (7–22)	**–**
Survival of PICU stay; *N* (%)	72 (77)	**–**
*Age-related subgroups*	*N* * (%)*	*Gender (M/F)*
Newborns (0–28 days)	33 (35)	22/11
Infants (1–12 months)	27 (29)	21/6
Children (1–6 years)	22 (24)	12/10
Children (6–18 years)	11 (12)	6/5

*IQR* interquartile range, *F* female, *M* male, *PICU* pediatric intensive care unit

Table 1 also shows the number of patients stratified into age-related subgroups and gender. There were more male patients in the age groups of newborns and infants, but the gender distribution between age-related subgroups was not significantly different (*p* > 0.05). There was an overall majority of males in this study and no significant difference of survival was recorded between male and female patients (77% vs. 78%; *p* > 0.05).

**Fig. 1:**
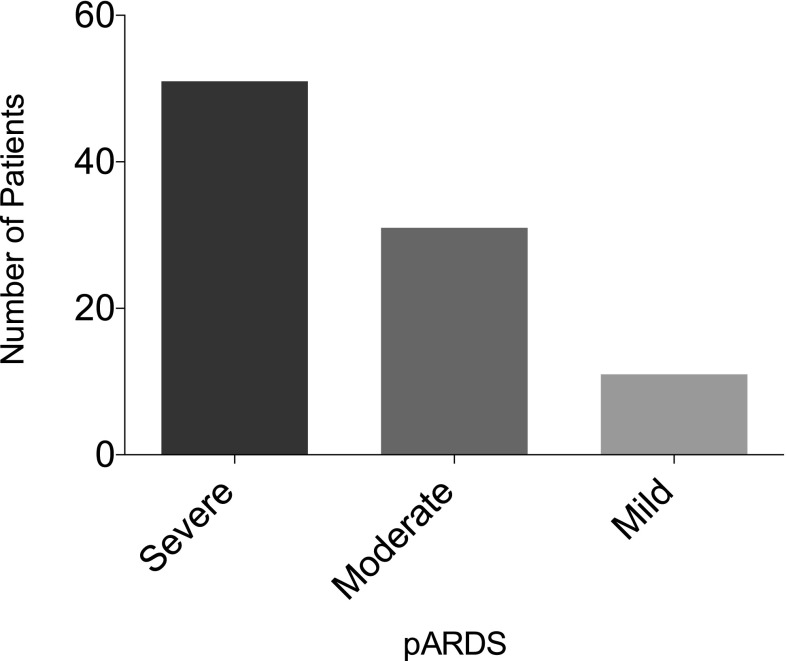
Severity of pediatric acute respiratory distress syndrome (*pARDS*). According to the modified Berlin Definition, most of the patients had severe pARDS on the day of admission

The median duration of PICU stay was 16 (10–27) days. In total, 66 patients (71%) had direct lung injury, 18 patients (19%) had indirect lung injury, and nine patients (10%) had lung injuries of unknown origin. No significant difference was found between the age-related subgroups concerning type of lung injury. Survival of patients with direct lung injury was 80% vs. 67% in patients with indirect lung injury. The survival of patients with direct lung injury was higher, but not significant (*p* > 0.05). The most common diagnosis leading to pARDS was viral pneumonia (21%), followed by nonspecified respiratory failure (16%) and bacterial pneumonia (15%). In 4% of all cases, the primary diagnosis was congenital heart defect (CHD). These patients developed pARDS additionally and secondary to their primary diagnosis of CHD during their PICU stay. The etiology of pARDS within the age-related subgroups is shown in Table 2. Viral pneumonia was among the most common causes of pARDS in infants (51%) and toddlers (23%). In children older than 6 years, bacterial pneumonia was the most common cause of pARDS with four patients (36%) presenting this diagnosis. The severity of pARDS according to BD on the day of admission is shown in Fig. 1. There was no significant difference in the survival rate or the duration of MV according to the severity of ARDS. Severity was also not significantly different among age-related subgroups (*p* > 0.05).

**Table 2:** Etiologies of pARDS

Diagnosis	Newborn (0–28 d)		Infant (1–12 mo)		Children (1–6 y)		Children (6–18 y)	
	(*N*)	(%)	(*N*)	(%)	(*N*)	(%)	(*N*)	(%)
MAS	10	31	–		–		–	
CDH	6	18	–		–		–	
Sepsis	5	15	3	11	3	14	1	9
Respiratory failure not specified	5	15	4	15	5	23	1	9
Bacterial pneumonia	2	6	4	15	4	18	4	37
Viral pneumonia	–		14	51	5	23	1	9
CHD	2	6	–		1	4	1	9
Lung bleeding	1	3	1	4	–		–	
Alveolar proteinosis	1	3	–		–		1	9
Asphyxia	1	3	–		–		–	
CF	–		1	4	1	4	1	9
Near drowning	–		–		3	14	1	9
Total	33	100	27	100	22	100	11	100

*d *days, *mo* months, *y* years, *MAS* meconium aspiration syndrome, *CDH* congenital diaphragmatic hernia, *CHD* congenital heart defect, *CF* cystic fibrosis

There were significant differences in the mode of ventilation used between the age-related subgroups (*p* < 0.001). Pressure-controlled ventilation was used in all age-related subgroups and was the leading mode of MV in patients older than 1 month. Noninvasive ventilation was used in two newborns and one school child. The median duration of MV was 12 days (7–22). The duration of MV was not significantly different among age-related groups.

**Fig. 2:**
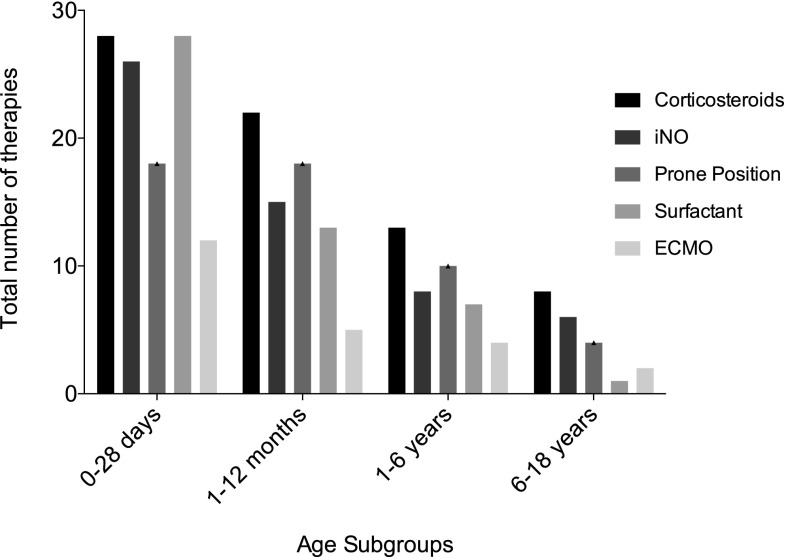
Supportive care treatment separated into age-related subgroups. The figure shows the distribution of treatment strategies between the four age subgroups. The distribution refers to the total number of the supportive therapies that were used and not to the number of patients. *ECMO* extracorporeal membrane oxygenation, *iNO* inhaled nitric oxide

Five types of supportive therapy options were investigated: iNO, surfactant, corticosteroids, prone positioning, and ECMO. Of all the patients, 75 (80%) received more than such treatment option. An overview of additional supportive treatments is listed in Table 3. A combination of three types of supportive therapies was most frequently applied to patients (*n* = 27, 29%). Eight patients (9%) received nonsupportive therapy beside MV and seven patients (7%) received all five supportive therapies. The impact of each single type of therapy was not analyzed separately. Despite the large number of different combinations of supportive treatment options, the combination of iNO, surfactant, corticosteroids, and prone positioning was the most common combination—used in 13 cases. Table 3 shows the overall frequency of supportive therapy applied to the study patients. The distribution of treatment strategies between the age-related subgroups is shown in Fig. 2. In total, 81% of all patients received more than one supportive therapy, and thus the distribution refers to the total number of supportive therapies used (and not to the number of patients). ECMO was used in 23 children, most of whom were <1 year. The median duration of ECMO was 11 days (7–16). The overall survival rate of patients who received ECMO was 52%.

**Table 3:** Supportive therapy options^a^

Supportive therapy	*N* (%)	Therapy options	*N* (%)
Corticosteroids	71 (76)	None	8 (9)
iNO	55 (59)	One	10 (11)
Prone position	50 (54)	Two	21 (23)
Surfactant	49 (53)	Three	27 (29)
ECMO	23 (27)	Four	20 (21)
		Five	7 (7)

*iNO* inhaled nitric oxide, *ECMO* extracorporeal membrane oxygenation

^a^Most of the patients (*N* = 76, 76%) received corticosteroids as supportive therapy beside mechanical ventilation. About every second patients was placed in prone position and treated with surfactant. Most of the patients had three different supportive therapies

## Discussion

This study evaluated pARDS patients and additional supportive treatments by using an age-related subgroup analysis. The first finding is that 66% of the 93 patients enrolled were male. This strong predominance of male gender was surprising since to date ARDS has not been reported to affect males more than females. Several studies on pARDS have shown male predominance but less pronounced—from 54 to 59% [2, 3, 14].

Patients were more frequently diagnosed with direct lung injury (71%) than indirect lung injury (19%). Patients with direct lung injury had an 80% survival rate, while the rate for indirect lung injury patients was 67%. Survival rates were not significantly different between these groups. This finding is in line with earlier pARDS studies, where the lowest survival rates were associated with sepsis, which is an indirect etiology of lung injury [14].

In this study, infection was the most common direct lung injury and viral pneumonia the most frequent diagnosis overall. Stratification by age subgroups showed that viral pneumonia is the most common diagnosis in infants (1–12 months) and among children (1–6 years), while children older than 6 years where affected by bacterial pneumonia. This finding confirms an earlier report by Wainwright [15].

The enrollment of newborns to pARDS studies is worthy of ongoing discussion. This period of life is characterized by a high risk of mortality [16], specifically among preterm newborns. Primary RDS related to prematurity is clearly different from pARDS in many aspects and consequently these patients were excluded. In full-term newborns, age specific-etiologies such as meconium aspiration and congenital diaphragmatic hernia serve as triggers for the development of pARDS. There is no clinical or biological evidence suggesting that lung injury in this group differs from older pARDS patients [17]. Since almost 36% of our patient collective were newborns and had pARDS, the management of these patients in our PICU plays an important role. Therefore, we decided to include full-term newborns like the other age groups into this study.

The severity of ARDS was defined according to BD. De Luca et al. reported that BD has improved validity for pARDS patients compared with the old AECC definition [13]. In this study, however, no significant differences were found in PICU survival or duration of MV measures. One reason might be the smaller number of patients compared with those in the study of De Luca et al., who analyzed 221 children in their multicenter study. In addition, the very heterogeneous patient collective presented here (patients ranged from newborns to children <18 years) differs from the study of De Luca et al., which focused on infants and toddlers aged >30 days and <18 months. Furthermore, the duration of MV was analyzed for all patients including those who did not survive. This might bias toward a shorter duration of MV (due to severely ill patients who died early in the course of treatment while being mechanically ventilated). The validity of this study’s results may be limited owing to the retrospective data collection and because levels of oxygenation were only collected at selected time points: namely, the day of admission, day 3, and day 7 of PICU stay.

To classify the severity of pARDS, the lowest oxygenation level of the admission day was used. Since most patients did not have arterial blood samples taken, the S/F ratio was used instead of the P/F ratio, according to the regression equation 1/SF = 0.00232 + 0.443/PF demonstrated by Khemani et al. This was used as a valuable surrogate for SpO_2_ levels between 80 and 97% [11]. All 93 patients included in the study were within this range.

The majority of this study’s patients received more than one supportive therapy. This explains why the impact of a single supportive therapy could not be analyzed. Furthermore, in this heterogeneous patient collective, those who did not receive one particular treatment would work poorly as a control group—the patient characteristics between the two patient groups are likely to show strong variety. However, one purpose of this study is its use as a basis for further prospective data collection where these limitations can be avoided.

The findings of the beneficial effects of the supportive treatment strategies investigated in this study are controversial. Although corticosteroids have been shown to improve the outcome of patients with meconium aspiration syndrome, data on the use of corticosteroids in pARDS are very limited [18]. Nevertheless, it was found to be the most frequently used supportive therapy in this study (76%). iNO was used in 59% of all our patients. The use of iNO has been reported to transiently improve oxygenation without improving patient outcome. iNO might be more effective in patients with very severe oxygenation deficit and in immunocompromised patients [19, 20]. Results concerning prone positioning showed improved oxygenation in children and adults with a tendency to lead to a survival benefit in patients with severe oxygenation deficit in adult studies [21, 22]. Prone positioning was the third most common supportive therapy (54%) for patients in this study. A recently published study shows that the response to prone positioning was variable in children with pARDS. Prone positioning improves the homogeneity of ventilation and may enhance recruitment of the dorsal lung regions [23].

More than 50% of patients in this study received surfactant as a supportive therapy; most of these patients were full-term newborns or infants. Similar findings are also reported in two studies where surfactant application was recommended especially in newborns [18, 24]. ECMO was also more frequently performed in newborns, showing a declining frequency of use with age. According to the ELSO Registry Report of 2016, the incidence of ECMO support has increased significantly in pediatric patients in general [25]. Recently published information on the ECMO survival rate of pediatric patients based on ELSO registry data showed a rate of 57%—slightly higher than the ECMO survival rate of 52% noted in this study [25]. However, in seven of the 23 ECMO patients, renal failure was present—something that has been reported as a contributing factor to reduced ECMO survival [26].

This study represents a valuable report on pediatric patients with ARDS and allows for an important extension of the application of the modified BD in all age groups. Moreover, this report suggests that the impact of therapeutic supportive therapies can be improved in the future by prospective data collection.

## Abbreviation

ARDS: acute respiratory distress syndrome
pARDS: pediatric acute respiratory distress syndrome
AECC: American–European Consensus Conference
BD: Berlin Definition
ECMO: extracorporeal membrane oxygenation
iNO: inhaled nitric oxide
PICU: pediatric intensive care unit
PaO_2_: arterial partial pressure of oxygen
SpO_2_: peripheral oxygen saturation
FiO_2_: fractional inspired oxygen concentration
MV: mechanical ventilation
CHD: congenital heart defect
